# Sociodemographic factors associated with multiple cardiovascular risk factors among Malaysian adults

**DOI:** 10.1186/s12889-015-1432-z

**Published:** 2015-01-31

**Authors:** Sumarni Mohd Ghazali, Zamtira Seman, Kee Chee Cheong, Lim Kuang Hock, Mala Manickam, Lim Kuang Kuay, Ahmad Faudzi Yusoff, Feisul Idzwan Mustafa, Amal Nasir Mustafa

**Affiliations:** Institute for Medical Research, Kuala Lumpur, Malaysia; Institute for Public Health, Kuala Lumpur, Malaysia; Disease Control Division, Ministry of Health, Kuala Lumpur, Malaysia

**Keywords:** Adult, Prevalence, Cardiovascular disease, Sociodemographic correlates, Lifestyle

## Abstract

**Background:**

To determine the prevalence and sociodemographic correlates of multiple risk factors for cardiovascular disease (CVD) among Malaysian adults.

**Methods:**

We analysed data on 1044 men and 1528 women, aged 24–64 years, participants in the Non Communicable Disease Surveillance 2005/2006, a nationally representative, population-based, cross-sectional study. Prevalence of obesity, high blood pressure, dyslipidaemia, hyperglycemia, physical inactivity, smoking, risky drinking, low vegetable and fruit intake were determined and multivariable logistic regression was used to identify sociodemographic factors associated with having ≥3 of these cardiovascular disease risk factors.

**Results:**

The response rate was 84.6% (2572/3040). Overall, 68.4% (95% CI: 63.2, 73.1) had at least three risk factors. Among men, older age and Indian ethnicity were independently associated with having ≥3 CVD risk factors; while among women, older age, low education, and housewives were more likely to have ≥3 CVD risk factors.

**Conclusion:**

The prevalence of cardiovascular risk factors clustering among Malaysian adults is high, raising concerns that cardiovascular disease incidence will rise steeply in the near future if no immediate preventive measures are taken. The current national health education and promotion programmes pertaining to modifiable risk factors can be further improved by taking into account the sociodemographic variation in CVD risk factors clustering.

## Background

Cardiovascular disease remains one of the most important chronic diseases in developed and developing countries. Globally, an estimated 17.3 million people died from cardiovascular disease (CVD) in 2008 alone, by 2030 this figure is estimated to reach 23.6 million [1]. Although CVDs have decreased in developed countries, the trend is increasing in developing nations [2]. In 2010, 25.4% (11812) of deaths in Malaysian government hospitals were due to cardiovascular diseases and it is also the top cause of premature death, with about 35% of deaths from CVD occurring in individuals aged below sixty [3].

Biological factors like hypertension, hyperglycaemia, hypercholesterolaemia, obesity and lifestyle factors [4] such as physical inactivity, smoking, excessive alcohol consumption and unhealth dietary behavior are established risk factors for cardiovascular disease. Increase in the prevalence of these risk factors is closely related to the rising prevalence of cardiovascular disease. Having multiple risk factors is associated with even greater risk for cardiovascular diseases as compared to having fewer risk factors [5]. Therefore the prevalence of multiple risk factors gives a clearer depiction of the burden of CVD risk in the population. From the public health perspective, knowledge on the true burden of CVD risk will help in devising appropriate action in prevention, detection and treatment of those with multiple CVD risk factors and ultimately curbing CVD in the population.

The presence of multiple risk factors have been previously reported, however, biological risk factors [6,7] and lifestyle risk factors [8,4] have been reported separately even though a person may have both types of factors concurrently. In this study we determined the prevalence of both biological and lifestyle risk factors in the adult Malaysian population. It is also important to elucidate the sociodemographic characteristics of those at higher risk of having multiple CVD risk factors. Therefore, we examined the sociodemographic factors that are associated with multiple CVD risk factors.

## Methods

### Data

The data used in this study was derived from the Malaysia Non-Communicable Disease Surveillance-1 (MyNCDS-1) survey. The MyNCDS-1 was a cross-sectional, population-based baseline survey on non-communicable diseases and its risk factors conducted in 2005 and 2006 [9]. Malaysia is administratively divided into 13 states and 3 federal territories. Participants were recruited from all thirteen states and one of the federal territories (Kuala Lumpur) through a complex, multistage cluster sampling design using the year 2000 national household sampling frame with the assistance of the Malaysian Department of Statistics. Stratifying variables were state/federal territory and setting (urban/rural), with enumeration blocks (EBs), living quarters (LQs) and households as the primary, secondary, and elementary sampling units respectively. The numbers of EBs and LQs selected per state were based on the desired sample size and proportionate to the 2005 Malaysian adult (age 25–64 years) population size for each state. In all, a total of 398 EBs and 1683 LQs were selected. All household members in all households in the selected LQs who met the eligibility criteria were included in the sample. A minimum required sample size of 2533 calculated based on prevalence of obesity of 5%, precision of 1.2% and design effect of 2. The final sample size of 3040 was computed after adding 20% expected non-response (507). In this paper, we analysed data comprised of 2572 subjects (1044 men and 1528 women) which is the final sample after removing subjects with incomplete information, yielding an estimated response rate of 84.6%. This final sample size exceeded the minimum required sample size (2533), therefore we assume that the missing data has minimal effect on the generalisability of the findings and has little effect on the relationship between sociodemographic factors and multiple cardiovascular risk factors. The details of the study design and sampling methods has been reported elsewhere [9]. The MyNCDS-1 survey was approved by the Medical Research and Ethics Committee of the Ministry of Health, Malaysia and is registered with the National Medical Research Register, National Institutes of Health, Ministry of Health of Malaysia (NMRR-13-1128-18447).

### Data collection

Collection of data was carried out simultaneously in all states from September 2005 to February 2008. An extensive field work manual was used as a practical guide for training sessions and subsequently during data collection in the field. Data collection was performed by trained field survey personnel consisting of nurses, health inspectors and research assistants under the supervision of medical doctors. The selected households were visited and eligible members of the household were interviewed personally upon giving informed consent. The interview was conducted using a structured questionnaire printed in English or Malay language (The questionnaire consisted of sections on sociodemographic characteristics, medical history, physical activity, smoking, alcohol consumption and dietary pattern). Later, appointments were made for physical examination (blood pressure, pulse rate, waist, height, weight and hip measurements) and biochemicals (glucose and lipid profile) measurement at a selected government health clinic nearest to the respondent’s residential area. All selected households were visited, those who were not at home during the visit were visited again at least two times before being classified as non-responders.

### Sociodemographic variables

Selected sociodemographic data on all the study subjects were analysed. The variables included gender, residential area (urban or rural), ethnicity, age, marital status, education, occupation, and monthly household income.

### Cardiovascular risk factors

#### Obesity

Obesity refers to general and/or abdominal obesity. Data on height and weight measurements were taken. Height was measured without footwear to the nearest 0.1 centimetre using a stadiometer. Weight was measured to the nearest 0.1 kilogram using a balance beam scale or SECA beam scale with minimal clothing and no shoes. Body mass index (BMI) (Weight [cm] /height^2^ [m]) was calculated and general obesity was defined as BMI ≥ 30.0 kg/m^2^. Waist circumference (WC) was measured directly over skin or over light clothing to the nearest 0.1 cm at the smallest circumference below the rib cage and above the umbilicus while standing with abdominal muscles relaxed. Abdominal obesity was defined as waist circumference ≥ 90 cm for men and ≥ 80 cm for women [10].

#### Hypertension

Blood pressure was measured by the auscultatory method [11] two or three times (if the first two readings differed by more than 10 mmHg), at no less than 30 seconds between measurements, and averaged. Hypertension was defined as having average systolic pressure ≥ 140 mmHg and/or diastolic pressure ≥ 90 mmHg or known case of hypertension [12].

Five ml of venous blood samples after overnight fasting were collected for the measurement of total cholesterol, HDL-cholesterol (HDL-C), triglycerides and glucose levels. It was placed into two vacuum test tubes; 2 ml blood into a test tube with NAF oxalate anticoagulant for blood sugar measurement and another 3 ml blood was filled into the test tube without anticoagulant for lipid profile. All the test tubes were properly labelled with respondents identification and date of blood collection. All blood-taking procedures was carried out under aseptic technique using a 5 ml syringe with 0.55 mm (21G) needle. All blood samples were transported in a cool box packed with dry ice to the central coordinating centre before being sent to the laboratory. All biochemical measurements were carried out according to the standard protocol of the WHO STEPwise approach to chronic disease risk factor surveillance [13].

#### Fasting lipid levels

The concentrations of HDL-cholesterol and triglycerides were measured using enzymatic assay kits (Automated HDL Cholesterol Flex® reagent cartridge and Triglyceride Flex® reagent cartridge). Serum total cholesterol was determined using enzymatic colorimetric tests with cholesterol esterase, cholesterol oxidase and glycerol phosphate oxidase respectively. Respondents were classified as having hypercholesterolemia if their total cholesterol was more than 5.2 mmol/L or were known cases of dyslipidemia or hypercholesterolemia [14]. Fasting plasma triglyceride >2.3 mmol/L was used as the cut-off point for presence of hypertriglyceridemia [14].

#### Diabetes mellitus

Those with no known diabetes were screened for diabetes mellitus using the two-hour post prandial glucose tolerance test. Glucose levels were measured using an enzymatic assay kit (Glucose Flex® reagent cartridge). Both those with fasting plasma glucose ≥7.0 mmol/L [15] or known case of diabetes mellitus were classified as diabetes mellitus in our study.

#### Inadequate physical activity

Physical activity was assessed using the Global Physical Activity Questionnaire (GPAQ) recommended by the WHO STEPwise approach to chronic disease risk factor surveillance [16]. The amount of energy accumulated from work, travelling and leisure time-related activities were quantified in terms of Metabolic Equivalent to Task (METs) minutes per week. One MET is defined as 1 cal/kg/hour or 3.5 ml/kg/min oxygen consumed, which is equivalent to sitting quietly. Accumulation of less than 600 METs per week is considered as being physically inactive [16].

#### Inadequate vegetable and fruit intake

Vegetable and fruit intake refers to consumption of all types of vegetables and fruits whether raw, cooked, dried or frozen. A serving of fruit is defined as one medium piece or two small pieces of fruit or one cup of diced pieces, a serving of vegetables is defined as half cup cooked vegetables or one cup of salad vegetables. Inadequate intake refers to consumption of less than five servings of vegetables and/or fruits daily [13].

#### Smoking

Smoking refers to current smoking which was defined as smoking any tobacco product daily at least once a day (Daily smoker), or smoked but not every day (Occasional smoker) at the time of the survey [17].

#### Risky drinking

Risky drinking is defined as consuming ≥14 standard alcoholic drinks per week for men and ≥7 standard drinks per week for women [18].

#### Multiple cardiovascular risk factors

Multiple cardiovascular risk factors were defined as having three or more cardiovascular risk factors whether biological (obesity, hypertension, hypertriglyceridaemia, hypercholesterolemia, Type II diabetes mellitus) or lifestyle (physical inactivity, smoking, risky drinking, inadequate fruit and vegetable intake).

### Statistical analyses

We described the sociodemographic characteristics of the study sample, the prevalence of each CVD risk factor and prevalence of having one to nine CVD risk factor/s by gender. Multivariable logistic regression was conducted separately for men and women to determine socio-demographic factors associated with clustering of ≥3 CVD risk factors. All the analyses were performed using SPSS software version 19.0 (SPSS Inc, Chicago). Sample weights were used in the analysis to adjust for the possible differences in the probability of EB and LQ selection or by non-response at the subjects’ level. Post stratification weights which took into account the population locality, gender and age-group stratification in 2005 were also applied.

## Results

The sociodemographic characteristics of the study participants are presented in Table 1. A majority of the respondents were Malay (55.4%), married (86.7%), with income less than RM1000 (Approximately USD290). Half of the female respondents were housewives.

**Table 1:** **Sociodemographic characteristics of respondents**

**Characteristics**	**Overall (n = 2572)**		**Men (n = 1044)**		**Women (n = 1528)**	
	**n**	**%**	**n**	**%**	**n**	**%**
Residential area						
Rural	1277	49.7	534	51.1	743	48.6
Urban	1295	50.3	510	48.9	785	51.4
Ethnicity						
Malay	1425	55.4	580	55.6	845	55.3
Chinese	461	17.9	186	17.8	275	18.0
Indian	231	9.0	80	7.7	151	9.9
Others	455	17.7	198	19.0	257	16.8
Age group						
25-34	610	23.7	229	21.9	381	24.9
35-44	738	28.7	277	26.5	461	30.2
45-54	747	29.0	313	30.0	434	28.4
55-64	477	18.5	225	21.6	252	16.5
Marital status						
Single	215	8.4	108	10.3	107	7.0
Married	2229	86.7	917	87.8	1312	85.9
Divorced/widowed	128	4.9	19	1.8	109	7.2
Education attainment						
No formal education	257	10.0	66	6.3	191	12.5
Primary school	778	30.2	316	30.3	462	30.2
Secondary school	1203	46.8	521	49.9	682	44.6
Tertiary	334	13.0	141	13.5	193	12.6
Occupation						
Public sector worker	266	10.3	157	15.0	109	7.1
Private sector employee	565	22.0	345	33.0	220	14.4
Self employed	627	24.4	399	38.2	228	14.9
Housewife	822	32.0	-	-	822	53.8
Others	292	11.3	143	13.7	149	9.8
Monthly income level						
Low (<RM 1000)	1206	48.8	483	46.8	723	50.2
Middle (RM 1000-RM3999)	1085	43.9	467	45.3	618	42.9
High (≥RM 4000)	180	7.3	81	7.9	99	6.9

The estimated prevalence of ≥3 CVD risk factors was 62.5% (95% CI: 58.5, 66.3), 64.3% (95% CI: 57.7, 70.4) among men and 60.6% (95% CI: 56.9, 64.2) among women. 1.5% had no risk factor (95% CI: 0.8, 2.7) 10.5% (95% CI: 8.7, 12.1) had biological risk factors only, 16.8% (95% CI: 14.9, 18.9) had lifestyle risk factors only and 71.5% (95% CI: 68.4, 74.3) had at least one biological and one lifestyle risk factor.

Among the biological risk factors, hypercholesterolemia (53.5% (95% CI: 47.3, 59.7)) and obesity (48.8 (95% CI: 45.4, 52.2)) were the most common, while the lifestyle risk factor with the highest prevalence was inadequate vegetable and fruit intake (72.8 (95% CI: 9.5, 75.9)) followed by physical inactivity (41.3 (95% CI: 37.4, 45.3)). Smoking and risky drinking were significantly higher among men while physical inactivity and obesity were significantly higher among women (Table 2).

**Table 2:** **Overall and gender-specific prevalence of cardiovascular risk factors**

**Characteristics**	**Overall**	**Men (n = 1044)**	**Women (n = 1528)**
	**% (95% CI)**	**% (95% CI)**	**% (95% CI)**
Biological			
Hypercholesterolemia	53.5 (47.3, 59.7)	53.2 (44.7, 61.5)	53.9 (49.2, 58.5)
General and/or abdominal obesity	48.8 (45.4, 52.2)	40.9 (36.3, 45.8)	57.2 (53.4, 60.9)
Hypertriglyceridemia	27.8 (20.8, 36.0)	31.9 (26.4, 38.1)	23.3 (14.7, 34.8)
Hypertension	25.8 (23.2, 28.6)	26.2 (22.5, 30.2)	25.4 (22.5, 28.5)
Diabetes mellitus	11.0 (9.2, 13.2)	9.8 (7.3, 12.9)	12.4 (10.6, 14.4)
Lifestyle			
Inadequate vegetable and fruit intake	72.8 (69.5, 75.9)	70.3 (65.6, 74.5)	75.5 (72.4, 78.4)
Inadequate physical activity	41.3 (37.4, 45.3)	36.8 (32.9, 40.8)	46.2 (41.0, 51.4)
Smoking	25.5 (23.0, 28.2)	46.5 (42.3, 50.8)	3.0 (2.1, 4.3)
Risky drinking	0.7 (0.3, 1.6)	1.2 (0.5, 3.1)	0.1 (0.02, 0.5)
Total number of biological and/or lifestyle risk factors			
0	1.5 (0.8, 2.7)	1.3 (0.5, 3.3)	1.8 (1.1, 2.9)
1	10.5 (8.6, 12.7)	9.9 (7.4, 13.3)	11.1 (8.4, 14.3)
2	25.5 (22.3, 29.0)	24.5 (19.8, 30.0)	26.6 (24.0, 29.3)
3	26.2 (23.4, 29.2)	24.7 (20.8, 29.1)	27.8 (24.7, 31.2)
4	20.9 (19.0, 23.0)	22.5 (19.0, 26.4)	19.2 (16.8, 22.0)
5	10.2 (8.8, 11.7)	10.8 (8.8, 13.2)	9.5 (7.7, 11.5)
6	3.8 (3.1, 4.6)	4.1 (3.0, 5.6)	3.4 (2.6, 4.5)
7	1.4 (0.9, 2.0)	2.0 (1.3, 3.2)	0.6 (0.4, 1.1)
≥3 biological/lifestyle risk factors	62.5 (58.5, 66.3)	64.3 (57.7, 70.4)	60.6 (56.9, 64.2)

Among men, the odds of having ≥3 CVD risk factors were higher among Indians and those aged ≥45 years. Among women, ≥3 CVD risk factors were more likely to be present among housewives, age ≥35 and with low education attainment (Table 3).

**Table 3:** **Prevalence and odds ratios for ≥3 cardiovascular risk factors by sociodemographic factors**

**Sociodemographic factor**	**≥3 cardiovascular risk factors**					
	**Men (n = 1044)**			**Women (n = 1528)**		
	**n**	**% (95% CI)**	**Adjusted OR (95% CI)**	**n**	**% (95% CI)**	**Adjusted OR (95% CI)**
Age group						
25-34	130	53.6 (43.7, 63.2)	Reference	173	42.8 (36.5, 49.3)	Reference
35-44	180	62.0 (52.1, 71.1)	1.25 (0.77, 2.02)	271	62.2 (56.8, 67.4)	1.81 (1.29, 2.34)*
45-54	246	75.4 (68.2, 81.5)	2.43 (1.53, 3.84)**	315	74.5 (68.3, 79.8)	3.10 (2.18, 4.41)**
55-64	178	79.8 (72.9, 85.3)	2.68 (1.39, 5.18)*	214	83.1 (76.7, 88.0)	5.76 (3.63, 9.15)**
Ethnicity						
Malay	416	62.1 (52.0, 71.2)	Reference	544	58.4 (53.4, 63.3)	Reference
Chinese	132	69.5 (60.2, 77.4)	1.47 (0.85, 2.52)	166	62.2 (54.1, 69.7)	0.93 (0.61, 1.41)
Indian	64	80.8 (68.2, 89.2)	2.85 (1.31, 6.23)*	100	68.4 (59.4, 76.2)	1.43 (0.82, 2.50)
Others	122	59.2 (51.5, 66.5)	0.89 (0.55, 1.43)	163	60.8 (53.7, 67.5)	1.06 (0.74, 1.54)
Marital status						
Single	63	57.3 (47.7, 66.4)	Reference	54	45.5 (35.1, 56.4)	Reference
Married	658	65.1 (57.7, 71.8)	1.02 (0.56, 1.83)	842	61.2 (57.1, 65.3)	0.91 (0.51, 1.62)
Divorced/widowed	13	71.1 (42.1, 89.3)	1.09 (0.29, 4.18)	77	70.9 (59.1, 80.5)	1.24 (0.60, 2.60)
Income level						
High (≥RM4000)	56	62.2 (47.1, 75.2)	Reference	62	59.4 (42.1, 74.7)	Reference
Middle (RM1000-3999)	339	63.3 (53.9, 71.8)	0.97 (0.46, 2.08)	381	58.4 (53.9, 62.7)	0.75 (0.38, 1.48)
Low (<RM1000)	329	65.3 (58.7, 71.3)	0.85 (0.34, 2.12)	466	60.7 (56.2, 65.0)	0.68 (0.32, 1.45)
Occupational status						
Private sector employee	235	60.9 (51.9, 69.2)	Reference	112	44.7 (37.1, 52.5)	Reference
Self-employed	268	61.3 (52.3, 69.6)	0.85 (0.58, 1.23)	141	60.3 (49.9, 69.8)	1.29 (0.79, 2.10)
Housewife	-	-	-	576	70.2 (66.2, 73.9)	2.16 (1.49, 3.14)**
Government employee	115	67.1 (57.8, 75.2)	1.50 (0.90, 2.50)	65	51.2 (38.8, 63.3)	1.24 (0.65, 2.38)
Others	116	78.6 (68.1, 86.3)	1.91 (0.87, 4.21)	79	46.2 (36.1, 56.7)	0.69 (0.34, 1.43)
Education status						
Tertiary	86	51.3 (33.4, 68.9)	Reference	91	39.0 (29.6, 49.3)	Reference
Secondary	373	64.7 (58.5, 70.4)	2.05 (0.89, 4.70)	418	58.6 (53.8, 63.2)	1.78 (1.13, 3.17)*
Primary	227	69.8 (62.8, 75.9)	1.97 (0.86, 4.52)	323	71.6 (66.6, 76.2)	1.91 (1.16, 3.17)*
No formal education	48	73.8 (57.6, 85.3)	2.58 (0.82, 8.18)	141	70.2 (61.8, 77.5)	1.97 (1.08, 3.59)*
Locality						
Urban	364	63.3 (53.6, 72.1)	Reference	490	60.1 (55.1, 65.0)	Reference
Rural	370	66.0 (61.0, 70.7)	1.32 (0.83, 2.10)	483	61.5 (57.1, 65.7)	0.98 (0.73, 1.32)

Adjusted OR, adjusted for all other variables in the model; *p-value < 0.05; **p-value <0.001.

## Discussion

The results suggest that taking into account various cardiovascular risk factors both biological and lifestyle, a vast majority (62.5%) of Malaysian adults would have three or more risk factors for cardiovascular disease. Data from the International Collaborative Study of Cardiovascular Disease in Asia (InterAsia), a cross-sectional nationwide survey conducted between 2000–2001, was analysed to determine the prevalence of CVD risk factor clustering among Chinese adults age 35–74 years old. They reported that 17.2% of Chinese adults age 35 to 74 had at least three of the following risk factors: dyslipidemia, hypertension, diabetes, smoking and overweight. In addition, they also reported 35.9% of US adults had these risk factors using data from the National Health & Nutrition Examination Survey of 1999–2000 [19]. The inclusion of more risk factors in our study may have contributed to the higher prevalence of multiple risk factors in our study than in China and US adults.

Data from the 1996 Malaysian National Health and Morbidity Survey showed that 61% of Malaysian adults age 30 and above had at least one cardiovascular risk factor and 27% had at least two or more risk factors out of four CVD risk factors investigated (hypertension, abnormal glucose tolerance, hypercholesterolemia and overweight) [20]. Selvarajah et al. [7] reported only 14% prevalence of ≥3 out of 4 biological risk factors (hypertension, hyperglycaemia, hypercholesterolaemia, central obesity) among adults age 18 and above, based on data from the 2006 National Health and Morbidity Survey. In addition to fewer number of risk factors, these two studies did not include lifestyle risk factors. In fact, lifestyle risk factors such as smoking have been identified as independent factors for CVD. And these factors are very prevalent in this country, for instance, prevalence of smoking alone among male adults was 46.5% [21] and physical inactivity was 43.7% (50.5% among female and 35.3% among male adults) [22]. Therefore, if we overlook these lifestyle factors we might underestimate the burden of CVD risk factors.

Among men, the odds of having ≥3 CVD risk factors were higher among Indian men and age 45 and above. A high proportion (70.6%, data not shown) of male Indians were obese, therefore, obesity is the major contributor to the multiple CVD risk factors among Indians in our study. Likewise, the 2006 Malaysian National Health and Morbidity Survey reported Indians had the highest prevalence of abdominal obesity compared to Malays and Chinese [23]. Another Malaysian study reported a similarly high 68.8% prevalence of abdominal obesity and 44.8% metabolic syndrome among Indians, the highest across the ethnic groups [24]. The high prevalence among Indians suggests that other than behavioural and dietary factors, genetics may play a role [25]. A review comparing obesity-related diseases between South Asians and white Caucasians found that South Asians had higher body fat, high truncal, subcutaneous and intra-abdominal fat, and low muscle mass, were different in terms of biochemical parameters (hyperinsulinemia, hyperglycemia, dyslipidemia, hyperleptinemia, low levels of adiponectin and high levels of C-reactive protein), procoagulant state and endothelial dysfunction, from white Caucasians [26]. These findings suggest risk factors other than lifestyle behaviours may contribute to multiple CVD risk in the Indian population.

Previous studies have reported on the increase in the prevalence of diabetes, hypertension, dyslipidaemia and obesity with age [7,24,27]. This indicates that there is an increasing trend in risk factors as the population ages. Our data showed older age was associated with multiple risk factors in both men and women. But, among men, the odds of having ≥3 risk factors was higher from middle age and above, while among women, significantly higher prevalence was observed at a younger age group (35–44). A probable explanation for this disparity is women become more sedentary and obese at a younger age compared to men.

In women, among the occupation groups, multiple risk factors was more likely to be found among housewives. This may be attributed to higher prevalences of obesity (60.3%), low fruit and vegetable consumption (82.7%) and physical inactivity (57.5%) among housewives in our study (Data not shown). Housewives had significantly higher odds of abdominal obesity [23] and physical inactivity [22] in the 2006 National Health and Morbidity Survey. A cross-sectional study in a rural Malay population in Malaysia showed being unemployed or a housewife is associated with metabolic syndrome [27].

Lower socioeconomic status (SES) which consists of the trio of education, income and occupation, is usually correlated with poor health. Our data showed women with lower education had significantly higher prevalence of multiple CVD risk factors. In a population-based study of 11247 Australian adults aged ≥25 years conducted in 1999–2000, women with lower education were associated with hyperinsulinemia, hypertriglyceridemia, abdominal obesity and hypertension [28]. It is known that lower education is associated with poorer health because of their poorer access, utilisation and understanding of health information [22]. We presume that this is because women with lower education may lack knowledge on healthy food and of healthy lifestyle practices.

The nation’s rapid economic growth accompanied by technological advancement and urbanization have led to changes towards a lifestyle of convenience and luxury with smoking, increasingly sedentary lifestyle and unhealthy dietary practices. These are modifiable risk factors that contributed to the rise in cardiovascular diseases. There is a need to introduce more creative and innovative health education and promotion programmes that result in behavioural modification. The Ministry of Health of Malaysia has recommended workplace-based and community-based programmes to empower individuals at high risk or with chronic diseases to develop health literacy, take responsibility for their own health and be actively involved in promoting health in their community [29].

One limitation in this study is worthy of note, this being a cross-sectional study, as such a definitive causal association between risk factors such as obesity and physical activity is not possible. Our sample size is relatively small compared to other national health surveys, but it was nationally representative.

## Conclusion

The prevalence of cardiovascular risk factors clustering among Malaysian adults is high, raising concerns that cardiovascular disease incidence will rise steeply in the near future if traditional approaches that target single risk factors continue to be used. It is time that a comprehensive, integrated approach that target all CVD risk factors be developed especially for those at high risk (housewives, low-educated and the elderly).

## Abbreviations

CVD: Cardiovascular disease
WC: Waist circumference
BMI: Body Mass Index
HDL-C: High Density Lipoprotein-Cholesterol
MET: Metabolic Equivalent of Task
